# MiRAGE: mining relationships for advanced generative evaluation in drug repositioning

**DOI:** 10.1093/bib/bbae337

**Published:** 2024-07-22

**Authors:** Aria Hassanali Aragh, Pegah Givehchian, Razieh Moslemi Amirani, Raziyeh Masumshah, Changiz Eslahchi

**Affiliations:** Department of Computer and Data Sciences, Faculty of Mathematical Sciences, Shahid Beheshti University, Daneshjou Blvd, District 1, Tehran 1983969411, Iran; Department of Computer and Data Sciences, Faculty of Mathematical Sciences, Shahid Beheshti University, Daneshjou Blvd, District 1, Tehran 1983969411, Iran; Department of Computer and Data Sciences, Faculty of Mathematical Sciences, Shahid Beheshti University, Daneshjou Blvd, District 1, Tehran 1983969411, Iran; Department of Computer and Data Sciences, Faculty of Mathematical Sciences, Shahid Beheshti University, Daneshjou Blvd, District 1, Tehran 1983969411, Iran; Department of Computer and Data Sciences, Faculty of Mathematical Sciences, Shahid Beheshti University, Daneshjou Blvd, District 1, Tehran 1983969411, Iran; School of Biological Sciences, Institute for Research in Fundamental Sciences (IPM), Farmanieh Ave, Tajrish, District 1, Tehran 193955746, Iran

**Keywords:** drug repositioning, drug–disease association, recommender systems, negative sampling, random forest models, feature selection

## Abstract

**Motivation:**

Drug repositioning, the identification of new therapeutic uses for existing drugs, is crucial for accelerating drug discovery and reducing development costs. Some methods rely on heterogeneous networks, which may not fully capture the complex relationships between drugs and diseases. However, integrating diverse biological data sources offers promise for discovering new drug–disease associations (DDAs). Previous evidence indicates that the combination of information would be conducive to the discovery of new DDAs. However, the challenge lies in effectively integrating different biological data sources to identify the most effective drugs for a certain disease based on drug–disease coupled mechanisms.

**Results:**

In response to this challenge, we present MiRAGE, a novel computational method for drug repositioning. MiRAGE leverages a three-step framework, comprising negative sampling using hard negative mining, classification employing random forest models, and feature selection based on feature importance. We evaluate MiRAGE on multiple benchmark datasets, demonstrating its superiority over state-of-the-art algorithms across various metrics. Notably, MiRAGE consistently outperforms other methods in uncovering novel DDAs. Case studies focusing on Parkinson’s disease and schizophrenia showcase MiRAGE’s ability to identify top candidate drugs supported by previous studies. Overall, our study underscores MiRAGE’s efficacy and versatility as a computational tool for drug repositioning, offering valuable insights for therapeutic discoveries and addressing unmet medical needs.

## Introduction

Amidst the escalating demand for effective treatments across a spectrum of diseases, ranging from infectious diseases to cancers and rare conditions, the conventional drug discovery and development process has gradually lost its appeal, primarily due to its time-intensive and costly nature [1, 2]. Typically, the process from drug discovery to clinical implementation spans over a decade, with associated expenses ranging from $500 million to $2 billion or more. Yet, only a meager fraction ¡10% of newly developed drugs receive approval for clinical use. To address these challenges, the pursuit of new indications for existing drugs, a concept known as drug repositioning, has emerged as an economically feasible and time-saving strategy [3, 4]. This approach emphasizes that the study of drug–disease associations (DDAs) plays a crucial role, particularly when considering the impact of drug–drug associations [5, 6]. In this context, harnessing computational methods for drug repositioning holds immense promise in identifying potential DDAs by combing through vast datasets. This approach not only expedites the drug discovery pipeline but also enables a more streamlined design of clinical trials [7–9]. Key to this computational approach are methods such as k-nearest neighbor (KNN), random forest, naive Bayes, and advanced deep learning models like convolutional neural networks and recurrent neural networks, and machine learning-based prediction methods extract relevant features from the biological information of drugs and diseases. These features, including molecular structures, genetic expressions, or pharmacological properties, are critical for understanding complex biological associations and mechanisms. As a result, the accurate extraction and analysis of these features allow researchers to address the drug repositioning problem more effectively, speeding up development, reducing costs, and improving the likelihood of successful treatment outcomes. In essence, drug repositioning can be viewed as a binary classification problem within this framework [10–16]. For example, PREDICT integrates various drug–drug and disease–disease similarities as input for a logistic regression classifier, enabling the prediction of unknown DDAs [10]. Additionally, there are deep learning-based approaches, employing multilayer interconnected neural networks to elevate the original features of drugs and diseases into high-level representations [17–23]. However, these methods necessitate substantial training data and demand meticulous fine-tuning to optimize performance across varying datasets. For instance, DeepDR integrates diverse association matrices and employs a multimodal autoencoder to efficiently derive feature embeddings for entities. Furthermore, it harnesses a variational autoencoder to detect novel diseases [17]. HNet-DNN leverages a deep neural network to predict DDAs by utilizing features extracted from the drug–disease heterogeneous network [18]. HINGRL constructs heterogeneous information networks, leveraging graph representation learning techniques to glean feature representations imbued with biological information [19]. DRHGCN is a drug repositioning method that utilizes a graph convolutional network framework [20]. DRWBNCF introduces an approach for predicting DDAs by proposing a weighted bilinear graph convolution operation [21]. In 2024, AMDGT emerged for predicting novel drug associations, employing dual-graph transformer modules. This model combines the similarity of data and intricate biochemical information. By leveraging an attention-aware modality interaction architecture, AMDGT showcases a sophisticated framework for predictive modeling in pharmaceutical research [22]. Although deep learning methods exhibit considerable promise in DDA prediction, their efficacy hinges on access to vast amounts of training data. Moreover, their performance often necessitates meticulous fine-tuning to adapt to varying training datasets. Additionally, deep learning-based approaches are particularly susceptible to overfitting, especially when confronted with sparse input DDA networks. In contrast, network-based methods represent another approach to predicting DDAs. These methods typically entail constructing multiple networks comprising not only drugs and diseases but also other pertinent entities such as long noncoding RNAs, microRNAs, target proteins, and more. Within this category, some methods directly utilize networks to generate predictions, while others leverage networks to access features of drugs and diseases, with downstream classification algorithms executing the prediction task [24–26]. DDAGDL utilizes a geometric approach on a heterogeneous network, enabling the effective learning of smoothed features through the utilization of an attention mechanism. [27]. However, network-based methods often prioritize the construction of heterogeneous networks without fully considering the distinct intrinsic characteristics of different types of molecules. This oversight limits the ability to fully leverage the potential knowledge encoded within biological networks for precise drug repositioning.

To address these limitations, we propose MiRAGE, a novel model for DDAs prediction. MiRAGE is meticulously crafted through a series of steps. First, we generate similarity matrices based on diverse features for both drugs and diseases. Next, multiple distinct DDAs scoring are created, each derived from a different set of features. These varied scores are then seamlessly integrated, providing a holistic perspective on DDAs. Finally, we utilize a Random Forest classifier to predict DDAs, feeding the resulting vector comprising these associations scoring for each drug–disease pair into the classifier. In the next section, we delineate the requisite datasets and elucidate the intricacies of the MiRAGE method. Our results section presents an exhaustive comparison between the outcomes yielded by MiRAGE and those attained by alternative methodologies, utilizing six criteria for evaluation. Furthermore, in the section of case studies, by focusing on drug repositioning’s primary goal we introduced a new treatment for Parkinson’s disease and schizophrenia. Furthermore, the last section furnishes the conclusion alongside prospects for future endeavors.

## Materials and methods

**Figure 1:**
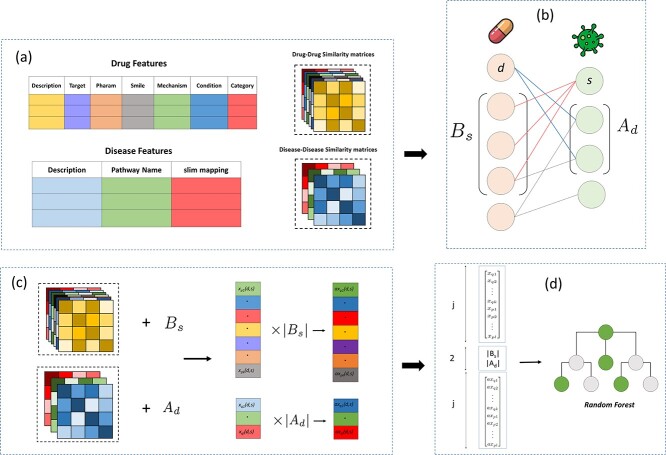
The scheme of MiRAGE workflow; (a) computing similarity matrices using Jaccard similarity for binary features and BERT similarity for text-based features; (b) utilizing associations mapping to construct sets of $A_{d}$ containing diseases associated with drug d and $B_{s}$ containing drugs associated with disease s; (c) obtaining association scoring and adjust association scoring between a drug d and a disease s based on each drug or disease feature, by leveraging the information of $A_{d}$ and $B_{s}$; (d) constructing a feature vector for a pair $(d,s)$ by concatenating these scoring and $|B_{s}|$, and $|A_{d}|$, and feeding it to a Random Forest model to predict the association between all drug and disease pairs.

In this section, we elaborate on MiRAGE, our advanced computational model designed for drug repositioning. The overall framework of MiRAGE is illustrated in Fig. 1.

### Problem formulation

Given a set of drugs $ D = \{d_{1}, \ldots , d_{n}\} $ and a set of diseases $ S = \{s_{1}, \ldots , s_{m}\} $, where n and m represent the total numbers of drugs and diseases, respectively. The task of drug repurposing can be defined as a function $ f: D \times S \rightarrow \{0, 1\} $, where 0 indicates no association and 1 indicates an association.

### Datasets

Our proposed model leverages four datasets: B-Dataset, C-Dataset, F-Dataset, and DDCD. The first three datasets are publicly available and frequently serve as benchmark datasets in various research papers, such as [19–22, 27]. The initial dataset, B-Dataset, extracted by Zhang *et al*. [28] from the CTD database, comprises 269 drugs identified with their DrugBank ID and 598 diseases identified with their OMIM ID, resulting in 18 416 known DDAs. The second dataset, C-Dataset, encompasses 2532 established associations between 663 drugs sourced from DrugBank and 409 diseases documented in OMIM [29]. The third dataset, F-Dataset, reported by Gottlieb *et al*. [10], contains 1933 known DDAs between 593 drugs collected from DrugBank and 313 diseases from OMIM. While they are valuable for this purpose, they are relatively small in size. Therefore, we collected another dataset with a significantly larger number of drugs and diseases, resulting in a higher number of associations. This dataset, termed the Drug Disease Comprehensive Dataset (DDCD), offers good accessibility and availability of features. The existing drugs in DDCD cover 90% of the drugs in F-Dataset, 96% of B-Dataset, and 88% of C-Dataset. The data for the DDCD were sourced from the Comparative Toxicogenomics Database (CTD), which is a valid and publicly available resource aimed at understanding the effects of environmental exposures on human health. CTD’s MEDIC disease vocabulary is a modified subset of descriptors from the “Diseases” category of the U.S. National Library of Medicine (NLM) Medical Subject Headings (MeSH); it includes 42 200 DDAs involving 1573 diseases identified by MESH IDs and 1410 drugs identified by DrugBank IDs. The diseases in DDCD are sourced from nearly 40 different categories, encompassing a wide range including “blood disease,” “cancer,” “cardiovascular disease,” “immune system disease,” “mental disorder,” and “nervous system disease,” among others. The diversity in both drugs and diseases within DDCD, along with the variability of features, holds significant potential for various applications and research endeavors, making it a strong candidate for designation as the gold standard dataset. For drug features, we utilized the DrugBank Database, recognized as one of the leading drug databases in the field. Disease features were sourced from the Comparative Toxicogenomics Database (CTD). The specific details about these four datasets can be found in Table S1 in the supplementary materials.

### Features description

Assume that we have a set of drugs and diseases features, denoted by $ f_{d} =\{p_{1}, \ldots , p_{k}\} $, where $ k $ is the total number of drug features, and $ f_{s} = \{q_{1}, \ldots , q_{l}\} $, where $ l $ is the total number of disease features. As shown in Fig. 1a, MiRAGE considers seven features from DrugBank for drugs information, including drug category, condition, description, mechanism of action (MOA), pharmacodynamics, SMILES representation, and target. The description is a text describing the key information of a drug, which is represented as the drug’s summary in DrugBank. Target refers to a list of interacting proteins, macromolecules, or small molecules identified with UniprotIDs. Pharmacodynamics involves studying a drug’s molecular, biochemical, and physiological effects, explaining its clinical or physiological workings. Isomeric SMILES is a widely used representation of a drug’s chemical structure. MOA describes how a drug functions at a molecular level. The condition represents a list of specific medical states for which a drug is indicated. Lastly, category entails therapeutic or general classifications like anti-convulsant or antibacterial. For the diseases, three features were gathered from CTD, including disease description, pathway name, and slim mapping. The disease description provides a concise definition and insight into each disease’s characteristics or symptoms; pathway names offer a brief list that represents molecular processes or interactions crucial for understanding disease development or progression; and slim mapping is a categorized list providing concise descriptors for the characteristics or affected systems of diseases. Each selected feature in our study has undergone careful consideration in collaboration with domain experts and holds distinct significance and value.

### MiRAGE method

In this section, we describe how MiRAGE identifies the association between a drug $ d $ and a disease $ s $. The process begins by collecting all diseases associated with $ d $ and all drugs associated with $ s $. For each drug and disease feature, we calculate the similarities between each pair of drugs, as well as between each pair of diseases. These obtained similarities are then fed into classifiers to predict the association between a given drug and disease. In the following text, we explain each step of the method in detail.

#### Construction of similarity matrices

Our goal is to describe the drug–drug and disease–disease similarities, aiding the identification of potential drug-repositioning candidates.

To analyze text-based features such as drug descriptions, MOA, pharmacodynamics, and disease descriptions, similarities are computed using the Bidirectional Encoder Representations from Transformers (BERT) model. This model serves the dual purpose of calculating similarities between these textual features and extracting embeddings, which are numerical representations, of the texts. These embeddings capture semantic information about the text, such as contextual meaning and relationships between words, by encoding them into dense vectors in a high-dimensional space. Subsequently, the similarities of embedding vectors are calculated using cosine similarity, a measure that quantifies the cosine of the angle between two vectors, providing a metric for their similarity.

For SMILES and categorical attributes such as Category, Condition, Target, Pathway Name, and slim mapping, Jaccard similarity is employed. Jaccard similarity is suitable for comparing one-hot encoding vectors, particularly in scenarios with very sparse vectors. The Jaccard similarity is defined as follows:


(1) \begin{align*} \text{Jaccard}(X_{di}, X_{dj}) &= \frac{|X_{di} \cap X_{dj}|}{|X_{di} \cup X_{dj}|} \end{align*}

Let us consider two feature vectors, $X_{di}$ and $X_{dj}$, each consisting of $n$ elements with values of either $0$ or $1$. $|X_{di} \cap X_{dj}|$ represents the count of common elements of $X_{di}$ and $X_{dj}$, while $|X_{di} \cup X_{dj}|$ denotes the count of elements in the union of $X_{di}$ and $X_{dj}$, respectively. Therefore, for each pair of drugs in each categorical drug feature, and each pair of diseases in each categorical disease feature, the similarity between the two will be calculated (Fig. 1a). Consequently, we will have $k$ similarity matrices, each with dimensions of $n \times n$, for our drug features, and $l$ similarity matrices, each with dimensions of $m \times m$, for our disease features.

#### Construction of associations vector score for drug–disease pairs

Suppose $ j = k + l $, where $ k $ and $ l $ are the number of features considered for drugs and diseases, respectively. For each drug–disease pair, we generate a feature vector of length $ 2j + 2 $, which serves as the input to a random forest model to predict the existence of an association between any given pair. To achieve this, for drug $ d $ ($ d \in D $) and disease $ s $ ($ s \in S $), we define $ A_{d} = \{s^{\prime} \mid s^{\prime} \sim d\} $, the set of all diseases originally associated with $ d $ in the training set, and $ B_{s} = \{d^{\prime} \mid d^{\prime} \sim s\} $, the set of all drugs originally associated with $ s $ in the training set (Fig. 1b). For each $ q $ in $ f_{s} $ and $ p $ in $ f_{d} $, four scores $ x_{q}(d, s) $, $ x_{p}(d, s) $, $ \text{ax}_{p}(d, s) $, and $ \text{ax}_{q}(d, s) $ are defined as follows:


(2) \begin{align*}\kern-2pt x_{q}(d,s) &= \max_{s^{\prime} \in A_{d}} \text{sim}_{q}(s, s^{\prime})\end{align*}

(3) \begin{align*} x_{p}(d,s) &= \max_{d^{\prime} \in B_{s}} \text{sim}_{p}(d, d^{\prime})\end{align*}

(4) \begin{align*}\kern-13pt \begin{aligned} \text{ax}_{\text{p}}(d,s) &= |A_{d}| \times \text{x}_{\text{p}}(d,s) \\ \text{ax}_{\text{q}}(d,s) &= |B_{s}| \times \text{x}_{\text{q}}(d,s) \end{aligned}\end{align*}

The rationale for using the maximum similarity rather than the mean or minimum in equations (2) and (3) is that a high similarity between s and even a single disease associated with d suggests a potential association. Similarly, if d is highly similar to just one of the drugs associated with s, it indicates a possible association.

Now, since we have k drug and l disease features, concatenating the scores $ x_{p}(d, s) $, $ x_{q}(d, s) $, $ \text{ax}_{p}(d, s) $, $ \text{ax}_{q}(d, s) $, $ |A_{d}| $, and $ |B_{s}| $ forms a vector of size $ 2j + 2 $ for the drug–disease pair $ (d, s) $ (Fig. 1c).

In this vector the scores $\text{ax}_{p}(d, s)$ and $\text{ax}_{q}(d, s)$ provide valuable information for the importance of each feature association. Indeed, multiplying $x_{p}(d, s)$ and $x_{q}(d, s)$ by the sizes of $A_{d}$ and $B_{s}$, respectively, we effectively weight these scores by the number of associations each drug or disease has. This weighting helps emphasize features that are more strongly supported by existing data, thereby improving the robustness and reliability of the model’s predictions.

Adding the sizes of $A_{d}$ and $B_{s}$ directly into the feature vector is also beneficial. These sizes provide additional information about the general connectivity or promiscuity of the drugs and diseases in the dataset. Highly connected drugs or diseases (those with many associations) might behave differently in terms of their predictability compared with those with fewer associations. Thus, including these sizes helps the model account for this variability, leading to more nuanced and accurate predictions.

#### DDA prediction

In this part of the method, we utilize the feature vectors of size $2j+2$ derived in the previous step for each drug–disease pair as input for a random forest model to perform binary classification (Fig. 1d). In this classification task, positive pairs denote instances in the training set where a drug is associated with a disease, while negative pairs represent drug–disease pairs assumed not to be associated. The random forest model endeavors to leverage these feature vectors to classify the positive and negative pairs in the training set with the highest accuracy and performance.

## Results

### Negative sampling

In training a random forest model for binary classification, obtaining labeled data with both positive and negative samples is essential. While positive samples can be extracted from known data sources, selecting suitable negative samples poses a challenge, particularly when the number of known data instances is much lower than that of unknown data. To address this issue, a method known as “hard negative mining” can be employed. Hard negative mining involves selecting negative samples from the pool of unknown data that are more likely to be misclassified as positive by the model. By focusing on these challenging negative samples, the model can learn to distinguish more effectively between positive and negative instances, ultimately improving its classification performance. The selection process for hard negative mining may involve various strategies, such as sampling from regions of feature space where positive samples are densely clustered or utilizing techniques like active learning to iteratively refine the selection of negative samples. In this study, we propose a three-step method for selecting suitable negative samples to address the challenge of imbalanced data in training a random forest model for binary classification. In this approach, the negative samples are generated using the unknown pairs, following the steps outlined below.

**First step:** We allocate 80% of the positive samples as the training set and the remaining 20% as the test set. This division remains constant throughout the process. Then, we divide the unknown pairs into $k$ segments, each containing approximately the same number of pairs as the positive samples. Similarly, we assign 80% of the negative samples from each segment as the training set and 20% as the test set within each segment. Next, we concatenate the training set of positive samples with the training set of negative samples from each segment. Similarly, we concatenate the test set of positive samples with the test set of negative samples from each segment.

**Second step:** The concatenated training and testing sets are fed into a classification model. By running the classification model, we determine the number of times each unknown pair is predicted as a positive sample. This count is recorded as the *num_pred* variable. This metric ranges from 0 to $k$, indicating how frequently each pair is classified as positive.

**Third Step:** We utilize all unknown drug–disease pairs with a *num_preds* value of zero as negative sampling pairs which employed for training our model.

### Ensuring classifier independence in negative sampling

To ensure that there is no dependency between MiRAGE and the classifier in the negative sampling method, we used Decision Tree and KNN for the negative sampling stage, and Random Forest as the final classifier of MiRAGE. The results of using these alternative classifiers for negative sampling on the F-Dataset are shown in Table S2. Similar results were obtained on the other datasets. We also experimented with various models for the final classification while keeping the negative sampling model as Random Forest. The detailed results of these experiments are discussed in the classification models section. The results obtained from these alternative models are very similar to the case in which we used Random Forest approaches in both methods. Additionally, these examinations ensure that there is no dependency between the classifiers chosen in MiRAGE and the negative sampling method.

### Statistical analyses of negative sampling selection

To demonstrate the significance of the negative sampling selection method, we compared the results of the MiRAGE method on the three datasets using both negative selection method and random negative selection. We selected random negative samples equal in number to our positive samples and run MiRAGE using these negative samples. This procedure was repeated 1000 times, each time with newly chosen random negative samples. We then compared the significance of the MiRAGE results with these randomized models across all metrics. The *P*-values for these comparisons are presented in Tables S3, S4, and S5. As shown in these tables, the consistently low *P*-values in all three datasets indicate that using the negative sampling approach significantly improves the results of MiRAGE compared with those obtained from random sampling.

### Classification models

In the final stage of the presented method, MiRAGE, we initially utilized the random forest model for classification. However, in this section, we explored various alternative classification models within the MiRAGE framework. These models encompass Logistic Regression, Decision Trees, Random Forests, KNN, and multi-layer perceptron (MLP). Our evaluation focused on assessing the impact of employing different classification methods within the MiRAGE algorithm. We conducted thorough evaluations using five-fold cross-validation techniques across all datasets. Our assessment incorporated six diverse metrics: AUROC, AUPR, Accuracy, precision, recall, and F1-score. The results on DDCD, F, B, and C datasets are presented in Table 1, Tables S6, S7, and S8 for each algorithm. The MLP-1 mentioned in these tables is a two-layer MLP with 32 units in the first layer and 64 units in the second layer. Both layers utilize the ReLU activation function, and the final layer’s activation function is sigmoid. MLP-2 is similar but slightly more complex, incorporating a third layer with 128 units and maintaining the same activation functions. These tables reveal that Random Forest consistently produces the highest scores across all metrics. Notably, Random Forest achieves exceptional performance with an AUROC of 99.98%, AUPR of 99.94%, Accuracy of 99.97%, Precision of 99.98%, Recall of 99.21%, and F1-score of 99.20%. We also evaluated the method under various parameter settings of the random forest model, on the F, B, and C datasets, to demonstrate its robustness, with the results presented in Tables S9, S10, and S11. So, in the subsequent sections of the paper, we focused exclusively on employing Random Forest classification models in the final step of the MiRAGE algorithm.

**Table 1:** Comparison of the results of using different classification models in MiRAGE algorithm on DDCD

**Model**	**AUROC**	**AUPR**	**Accuracy**	**Precision**	**Recall**	**F1-score**
LogisticRegression	0.9749	0.8877	0.9947	0.9952	0.8913	0.8566
KNN	0.9343	0.8561	0.9960	0.9944	0.9039	0.8908
Decision Tree	0.9884	0.8350	0.9961	0.9991	0.9884	0.9113
MLP-1	0.9867	0.9229	0.9969	0.9883	0.9318	0.9175
MLP-2	0.9888	0.9265	0.9969	0.9903	0.9304	0.9187
Random Forest	**0.9998**	**0.9994**	**0.9997**	**0.9998**	**0.9921**	**0.9920**

### Feature selection

In this section, we assessed the significance of the features utilized by the Random Forest model within the MiRAGE method. Feature importance in our Random Forest model is determined using the Gini importance (Mean Decrease in Impurity) method. This approach evaluates the decrease in node impurity (such as Gini impurity or entropy) each time a feature is used to split the data within the trees of the forest. The decreases in impurity are aggregated and averaged across all trees, providing a score that reflects the overall contribution of each feature to the model’s predictions. Features with higher importance values were deemed more influential in making predictions. In Table S12, we provided the feature importance of all 22 features. The three most important features based on these scores—Adjusted Drug Condition, Adjusted Disease Slim Mapping, and Adjusted Drug Category—are influential in drug repositioning for several reasons. The Adjusted Drug Condition feature captures the versatility and therapeutic potential of a drug across multiple diseases. This is crucial because a drug that is effective for a variety of conditions is more likely to be successfully repurposed. The Adjusted Disease Slim Mapping feature simplifies complex disease ontologies while retaining key characteristics, making it easier to identify patterns and similarities between diseases that can be targeted by similar drugs. This helps in pinpointing new therapeutic uses for existing drugs. Finally, the Adjusted Drug Category feature provides a high-level overview of a drug’s potential uses and effectiveness based on its therapeutic class, chemical structure, or MOA. This categorization helps in understanding which drug classes are more versatile and can be applied to multiple diseases, thus aiding in efficient drug repositioning. Each of these features, by capturing essential and broad aspects of drug and disease relationships, significantly enhances the model’s ability to predict new and effective DDAs. To evaluate our model’s performance, we iteratively incorporated features from the top to the end, monitoring six metrics to achieve MiRAGE’s results considering these features. Beginning with the top feature, we progressively added one feature at a time, training the model accordingly. This process continued until all 22 features were included. We recorded the results and evaluated the effectiveness of each feature subset for comparison. As shown in Table S13, which displays the outcomes of this process on DDCD, adding each feature improved the method’s results. Thus, we can conclude that all of these features are necessary for obtaining the best performance.

### Comparison with state-of-the-art algorithms

We implemented MiRAGE on the three benchmark datasets, F, B, and C, which establish associations between DrugbankID and OMIM-ID for drug-repositioning tasks. Leveraging this association, we utilized all of the drug features mentioned previously across the datasets. Since we lacked access to disease features associated with OMIM-ID, we turned to Phenotype similarity, as introduced by Junkai Liu *et al*. [22], as an alternative approach. The results were compared with five state-of-the-art methods: DRHGCN, HINGRL, DRWBNCF, DDAGDL, and AMDGT. The experimental results are reported in Tables 2, S14, and S15.

**Table 2:** The results of state-of-the-art and MiRAGE methods on the F-Dataset

**Model**	**AUROC**	**AUPR**	**Accuracy**	**Precision**	**Recall**	**F1-score**
DRHGCN	0.9207	0.9375	0.8583	0.9309	0.7739	0.8452
HINGRL	0.9366	0.9449	0.8645	0.8832	0.8402	0.8612
DRWBNC	0.8958	0.9200	0.8296	0.8752	0.8237	0.8341
DDAGDL	0.9239	0.9235	0.8513	0.8475	0.8567	0.8521
AMDGT	0.9598	0.9617	0.8905	0.8741	0.9128	0.8929
MiRAGE	**0.9985**	**0.9985**	**0.9920**	**0.9930**	**0.9907**	**0.9907**

Across all three datasets, MiRAGE consistently outperformed the other baseline methods in terms of AUROC, AUPR, Accuracy, Precision, Recall, and F1-score. Specifically, on the B-Dataset, MiRAGE achieved a notable 13% improvement in accuracy and F1-score. On the C-Dataset, our model demonstrated a 2% enhancement in accuracy and F1-score compared with the closest competitor, ADGMT, although ADGMT exhibited a higher recall score. Lastly, on the F-Dataset, MiRAGE demonstrated a remarkable 10% increase in accuracy and F1-score. These findings highlight MiRAGE’s exceptional performance across diverse datasets.

## Case studies

To assess MiRAGE’s efficacy in uncovering novel applications for drugs, leading to the development of new treatments for existing diseases, we particularly focused on drug repositioning’s primary goal. We conducted two key case studies centered around neurological and mental health disorders. Specifically, we selected Parkinson’s disease within the neurological domain and schizophrenia within the realm of mental health. The rationale behind these selections stems from the inadequacy of current treatments for both conditions.

We utilized our model on F-Dataset, which is a commonly employed case study dataset in various papers. Our approach involves assessing predicted associated probabilities for unlabeled pairs and arranging pair candidates in descending order of these probabilities. This ranking system enabled us to identify the pairs most likely to have an effect on the disease treatment, with the top-ranked pairs representing the highest likelihood of efficacy.

Subsequently, we identified the top seven drugs for the respective diseases, along with their supporting evidence presented in Table 3. To evaluate the proposed drugs, we conducted a thorough examination based on reliable previous studies specific to each pair. These studies were sourced from various journals, although many pairs had multiple studies available, and some had been updated with new purposes in recent association datasets.

**Table 3:** Top seven candidate drugs predicted by MiRAGE for Parkinson and schizophrenia on the F-Dataset

**Diseases**	**Candidate drugs**	**Evidences**
Parkinson	Bromocriptine	Lieberman *et al*. [30]
	Cabergoline	Pastor *et al*. [31]
	Zonisamide	Li *et al*. [32]
	Levodopa	Gandhi *et al*. [33]
	Biperiden	DrugBank
	Cyproheptadine	–
	Trihexyphenidyl	–
Schizophrenia	Methotrimeprazine	DrugBank
	Paroxetine	Wang *et al*. [34]
	Triflupromazine	Oliveira *et al*. [35]
	Gabapentin	Gabriel *et al*. [36]
	Oxcarbazepine	Popova *et al*. [37]
	Bromocriptine	–
	Azathioprine	–

Starting with Parkinson’s disease, the first candidate drug, Bromocriptine, is an ergo peptide derivative and dopamine agonist. The case study provided by Lieberman and Goldstein [30] concludes that low-dose bromocriptine therapy is effective, and is probably an alternative to levodopa as a drug of first choice in Parkinson’s disease. Even DrugBank references the positive effect of it on early Parkinson’s. The second-highest score was achieved by Cabergoline, another dopamine receptor agonist. Research conducted by Pastor and Tolosa [31] investigated its efficacy in Parkinson’s disease treatment, by improving motor symptoms. Zonisamide, placed as the third suggestion, is a sulfonamide anticonvulsant used to treat partial seizures; moreover, Li *et al*. [32] introduced it as an add-on treatment to overcome the deficiencies of the general therapies currently used. The next one is Levodopa, a dopamine precursor. Its positive effect is reported by Gandhi and Saadabadi [33]. The last one is Biperiden, which is introduced as a muscarinic receptor antagonist and has been mentioned as an effective treatment for Parkinson’s in the latest update of the DrugBank Database.

Schizophrenia, a mental health condition characterized by delusions, hallucinations, disorganized speech, and lack of motivation, often lacks efficient treatment options. However, our method has identified seven drugs with a high probability of effectiveness. First on the list is Methotrimeprazine, primarily used in managing manic phases of bipolar disorder. It is recognized in the latest update of DrugBank for managing psychosis, particularly in cases of schizophrenia. The second one is Paroxetine, a selective serotonin reuptake inhibitor used to treat major depressive disorder. Moreover, Wang *et al*.’s [34] study concludes that it can effectively relieve the symptoms of schizophrenia, and alleviate the depressive symptoms of patients, with high safety. Thus, it is worth promoting. The next one is trifluoperazine, a phenothiazine used to treat depression, anxiety, and agitation. According to a study by de Oliveira Marques *et al*. [35], it has been found effective at low doses for patients with schizophrenia. The fourth proposed drug is Gabapentin, an anticonvulsant medication, utilized for managing peripheral neuropathic pain. A study by the Departments of Psychiatry and Community Health Sciences at the University of Calgary suggests that it can be effectively used in cases of partially responsive schizophrenia [36]. The fifth one is an anti-epileptic, used in the treatment of partial-onset seizures named Oxcarbazepine. According to a study by Ekaterine Popova, it might also benefit patients with schizoaffective disorder who have not responded well to other treatments [37]. The last two proposed drugs are novel associations predicted by MiRAGE for Parkinson’s disease and schizophrenia. These associations have not been previously reported or experimented on, thereby highlighting MiRAGE’s potential to uncover novel therapeutic possibilities. Cyproheptadine and Trihexyphenidyl are suggested as novel drugs for Parkinson’s disease and Bromocriptine and Azathioprine for schizophrenia.

## Discussion

In this paper a similarity-based method due to its ability to leverage the concept that drugs and diseases with similar properties are likely to interact similarly is opted. This approach enhances the potential for effective repositioning by predicting that drugs with similar characteristics can be beneficial for treating diseases with similar properties. The MiRAGE method offers several advantages over other drug-repositioning methodologies. Our method boasts high flexibility, allowing for the customization of features, similarity methods, and classification models based on specific use cases. By meticulously curating distinct features that aptly encapsulate the nuanced characteristics of both drugs and diseases, our approach ensures a comprehensive assessment of potential associations. MiRAGE is designed to be fast and easy to run, making it accessible for researchers and practitioners without requiring extensive computational resources or specialized expertise. Our use of multiple datasets and comparisons with state-of-the-art methods demonstrates the robustness and superiority of MiRAGE. The superior performance of MiRAGE in predicting DDAs suggests that our method can more accurately identify potential drug repositioning opportunities, leading to a higher success rate in preclinical and clinical testing, and ultimately accelerating the drug discovery process. Moreover, one of the key advantages of MiRAGE is the explainability of its results. Each prediction can be traced back to specific features, providing transparency and allowing researchers to understand the underlying reasons for each DDA. This feature enhances trust in the model’s predictions and facilitates further investigation and validation. One of the application scopes of the developed method to benefit the real-world application is drug–gene prediction. Consider a set of drugs (D), a set of diseases (S), and a set of genes (G). There are known associations between drugs and diseases (D-S), and between diseases and genes (S-G). By identifying shared genes among diseases, we can use this information as one of the features and leverage these connections in any method that predicts drug–gene associations to improve its performance. While this approach offers significant advantages, it is important to acknowledge certain limitations. The effectiveness of the MiRAGE is contingent upon the availability of a large dataset comprising diverse drugs, diseases, and their respective associations. This requirement may pose challenges in scenarios where comprehensive data are limited, potentially impacting the method’s ability to generate accurate recommendations. Thus, while our method demonstrates considerable promise, expanding and enriching datasets will be essential to fully leverage its capabilities in drug discovery and repurposing efforts. Finally, while positive samples can be readily extracted from known data sources, identifying suitable negative samples becomes challenging, particularly when the number of known instances is significantly lower than that of unknown data. To tackle this issue, we have devised a novel approach that consistently yields superior results.

**Key Points**

- MiRAGE is an effective and versatile computational method for drug repositioning to identify new therapeutic uses for existing drugs.
- It utilizes a three-step framework including negative sampling using hard negative mining, classification employing random forest models, and feature selection based on feature importance.
- Effective integration of diverse drug and disease features within a random forest model results in favorable outcomes.

## Supplementary Material

SUPP-bbae337

## Data Availability

The source code and datasets are available at https://github.com/ARIASHA/MiRAGE.
