# Supplementary material for: MiRAGE: mining relationships for advanced generative evaluation in drug repositioning
Source: Brief Bioinform. 2024 Jul 22;25(4):bbae337. doi: 10.1093/bib/bbae337 (PMC11262809; doi:10.1093/bib/bbae337)
Supplement: SUPP-bbae337 [file supp-bbae337.pdf]

Table S1. Details of Datasets.

Dataset	Drugs	Diseases	Associations	Sparsity
B-Dataset	269	598	18,416	0.1144
C-Dataset	663	409	2,532	0.0093
F-Dataset	593	313	1,933	0.0104
DDCD	1,410	1,573	42,200	0.019

Table S2. Comparison of the outcomes when employing KNN and Decision Tree models for negative sampling in the MiRAGE algorithm on the DDCD, using Random Forest as the ultimate classifier.

Model	AUROC	AUPR	Accuracy	Precision	Recall	F1-score
KNN	0.9968	0.9964	0.9705	0.9719	0.9680	0.9652
Decision Tree	0.9997	0.9981	0.9982	0.9821	0.9978	0.9806

To demonstrate the significance of the results of our negative sampling on all three datasets we employed three different statistical tests on our findings. We selected random negative samples equal in number to our positive samples and trained a Random Forest model using these negative samples. This procedure was repeated 1000 times, each with newly chosen random negative samples. We then compared the significance of the MiRAGE results to these randomized models across all metrics. The p-values are reported in tables S3, S4, and S5. The very small number of p-values in all three data sets indicates that using the negative sampling approach significantly improves the results of MiRAGE compared to those obtained from random sampling.

Table S3. Statistical tests on the F-Dataset.

Hypothesis Test	AUROC	AUPR	Accuracy	Precision	Recall	F1-score
One Sample T-test	5.955e-90	3.267e-71	6.409e-109	2.924e-117	1.781e-75	8.238e-110
U-Test	5.640e-39	5.640e-39	5.423e-39	5.627e-39	5.060e-39	5.627e-39
Wilcoxon Test	3.897e-18	3.897e-18	3.781e-18	3.890e-18	3.584e-18	3.890e-18

Table S4. Statistical tests on the B-Dataset.

Hypothesis Test	AUROC	AUPR	Accuracy	Precision	Recall	F1-score
One Sample T-test	1.732e-101	3.405e-101	5.290e-101	1.935e-101	5.761e-95	6.599e-101
U-Test	7.116e-38	7.116e-38	7.099e-38	7.116e-38	7.086e-38	7.116e-38
Wilcoxon Test	2.746e-18	2.746e-18	2.741e-18	2.746e-18	2.732e-18	2.746e-18

Table S5. Statistical tests on the C-Dataset.

Hypothesis Test	AUROC	AUPR	Accuracy	Precision	Recall	F1-score
One Sample T-test	3.978e-120	1.591e-03	5.852e-98	9.227e-130	4.187e-112	9.877e-106
U-Test	5.640e-39	8.973e-05	5.406e-39	5.611e-39	3.683e-39	5.609e-39
Wilcoxon Test	3.896e-18	2.065e-03	3.772e-18	3.881e-18	2.746e-18	3.880e-18

Table S6. Comparison of the results of using different Classification Models in MiRAGE algorithm on the F-Dataset.

Model	AUROC	AUPR	Accuracy	Precision	Recall	F1-score
RandomForest	0.9985	0.9985	0.9920	0.9930	0.9907	0.9907
DecisionTree	0.9838	0.9715	0.9841	0.9838	0.9838	0.9816
KNN	0.9855	0.9835	0.9716	0.9742	0.9684	0.9664
LogisticRegression	0.9913	0.9929	0.9761	0.9776	0.9736	0.9719

Table S7. Comparison of the results of using different Classification Models in MiRAGE algorithm on the B-Dataset.

Model	AUROC	AUPR	Accuracy	Precision	Recall	F1-score
RandomForest	0.9996	0.9996	0.9918	0.9917	0.9918	0.9917
DecisionTree	0.9664	0.9438	0.9662	0.9663	0.9664	0.9660
KNN	0.9982	0.9974	0.9896	0.9920	0.9894	0.9894
LogisticRegression	0.9858	0.9884	0.9494	0.9475	0.9493	0.9482

Table S8. Comparison of the results of using different Classification Models in MiRAGE algorithm on the C-Dataset.

Model	AUROC	AUPR	Accuracy	Precision	Recall	F1-score
RandomForest	0.9733	0.9672	0.9209	0.9210	0.9209	0.9214
DecisionTree	0.8617	0.8086	0.8617	0.8619	0.8617	0.8633
KNN	0.9548	0.9369	0.9111	0.9112	0.9111	0.9119
LogisticRegression	0.9655	0.9654	0.9140	0.9144	0.9140	0.9120

We explored the sensitivity of our Random Forest model to variations in the number of estimators and the splitting criterion (Gini impurity vs. entropy). The number of estimators refers to the number of decision trees in the forest, while the splitting criterion determines the function used to measure the quality of a split. The results of these experiments are presented in Tables S9, S10, and S11 for the F, B, and C datasets. These results demonstrated consistently high performance across different parameter settings, underscoring the robustness and reliability of our model in predicting associations between drug-disease pairs.

Table S9. Comparison of Random Forest classifiers on the F-Dataset with different parameters.

Estimator	Criterion	AUPRC	AUROC	Precision	Recall	Accuracy	F1-score
300	gini	0.998577	0.998682	0.993097	0.990790	0.992045	0.990704
400	gini	0.998592	0.998663	0.993097	0.990790	0.992045	0.990704
500	gini	0.998528	0.998539	0.993097	0.990790	0.992045	0.990704
600	gini	0.998609	0.998629	0.993097	0.990790	0.992045	0.990704
700	gini	0.998705	0.998750	0.993097	0.990790	0.992045	0.990704
300	entropy	0.998577	0.998682	0.993097	0.990790	0.992045	0.990704
400	entropy	0.998592	0.998663	0.993097	0.990790	0.992045	0.990704
500	entropy	0.998528	0.998539	0.993097	0.990790	0.992045	0.990704
600	entropy	0.998609	0.998629	0.993097	0.990790	0.992045	0.990704
700	entropy	0.998705	0.998750	0.993097	0.990790	0.992045	0.990704

Table S10. Comparison of Random Forest classifiers on the B-Dataset with different parameters.

Estimator	Criterion	AUPRC	AUROC	Precision	Recall	Accuracy	F1-score
300	gini	0.999629	0.999596	0.991975	0.992063	0.992014	0.991877
400	gini	0.999644	0.999615	0.992108	0.992194	0.992147	0.992011
500	gini	0.999644	0.999619	0.991843	0.991927	0.991881	0.991740
600	gini	0.999648	0.999619	0.992107	0.992199	0.992147	0.992013
700	gini	0.999645	0.999616	0.992107	0.992199	0.992147	0.992013
300	entropy	0.999629	0.999596	0.991975	0.992063	0.992014	0.991877
400	entropy	0.999644	0.999615	0.992108	0.992194	0.992147	0.992011
500	entropy	0.999644	0.999619	0.991843	0.991927	0.991881	0.991740
600	entropy	0.999648	0.999619	0.992107	0.992199	0.992147	0.992013
700	entropy	0.999645	0.999616	0.992107	0.992199	0.992147	0.992013

Table S11. Comparison of Random Forest classifiers on the Random C-Dataset with different parameters.

Estimator	Criterion	AUPRC	AUROC	Precision	Recall	Accuracy	F1-score
300	gini	0.965731	0.973092	0.923928	0.923913	0.923913	0.924138
400	gini	0.967798	0.973549	0.921978	0.921937	0.921937	0.922321
500	gini	0.967201	0.973324	0.921008	0.920949	0.920949	0.921415
600	gini	0.966772	0.973236	0.922018	0.921937	0.921937	0.922473
700	gini	0.966426	0.973547	0.924048	0.923913	0.923913	0.924584
300	entropy	0.965731	0.973092	0.923928	0.923913	0.923913	0.924138
400	entropy	0.967798	0.973549	0.921978	0.921937	0.921937	0.922321
500	entropy	0.967201	0.973324	0.921008	0.920949	0.920949	0.921415
600	entropy	0.966772	0.973236	0.922018	0.921937	0.921937	0.922473
700	entropy	0.966426	0.973547	0.924048	0.923913	0.923913	0.924584

Table S12. The importance scores of each feature in MiRAGE algorithm on DDCD. We ensured a rigorous assessment of the significance of each feature used by the Random Forest prediction model. The importance of each feature was quantified based on the decrease in impurity, such as Gini impurity or entropy, resulting from splitting on that feature across all trees in the ensemble. Gini impurity measures the likelihood of an incorrect classification of a randomly chosen element if it was randomly labeled according to the distribution of labels in the dataset. Features with higher importance values were considered more influential in making predictions.

**Feature**	**Importance Score**
Adjusted Drug Condition	0.373817
Adjusted Disease Slim mapping	0.240405
Adjusted Drug Category	0.172189
Disease Description	0.025211
Drug Smile	0.020130
Disease Pathway Name	0.018714
Adjusted Disease Description	0.018105
Adjusted Disease Pathway Name	0.017654
Drug Pharmacodynamics	0.017114
Drug Description	0.012933
Drug Mechanism	0.012879
Drug Category	0.010547
Adjusted Drug Pharmacodynamics	0.009024
Adjusted Drug Smile	0.008870
Adjusted Drug Mechanism	0.007757
Adjusted Drug Description	0.007085
Adjusted Drug Target	0.006878
Drug Condition	0.005622
$\lvert B_s \rvert$	0.005010
$\lvert A_d \rvert$	0.004026
Drug Target	0.003735
Disease Slim mapping	0.002295

Table S13. The results of Top-K ranking features on DDCD. To evaluate the performance of MiRAGE, we employed an iterative process where features were incorporated progressively from the highest to the lowest importance value. Starting with the top feature, we added one feature at a time and retrained the model accordingly, monitoring six key metrics to assess the model's performance with each feature subset.This iterative process continued until all 22 features were included. Consequently, we concluded that all 22 features are necessary to achieve the best predictive accuracy and overall performance of MiRAGE.

Model	AUROC	AUPR	Accuracy	Precision	Recall	F1-score
top 1	0.847442	0.603530	0.987736	0.912419	0.742293	0.615990
top 2	0.949016	0.796130	0.991733	0.939322	0.838177	0.767988
top 3	0.951891	0.874053	0.994852	0.958351	0.906886	0.864668
top 4	0.970934	0.895163	0.996081	0.984626	0.914745	0.895127
top 5	0.978085	0.909981	0.996474	0.988851	0.920605	0.905918
top 6	0.982499	0.918974	0.996743	0.992775	0.924044	0.913113
top 7	0.982677	0.923813	0.996963	0.995053	0.926648	0.919122
top 8	0.983236	0.923991	0.996944	0.995553	0.924251	0.918515
top 9	0.999580	0.999284	0.999804	0.997173	0.995868	0.995141
top 10	0.999576	0.999300	0.999843	0.998920	0.996324	0.996108
top 11	0.999573	0.999301	0.999848	0.998923	0.996737	0.996236
top 12	0.999570	0.999297	0.999785	0.999390	0.994819	0.994662
top 13	0.999572	0.999322	0.999809	0.999600	0.995748	0.995248
top 14	0.999572	0.999328	0.999825	0.999763	0.996655	0.995638
top 15	0.999572	0.999329	0.999785	0.999390	0.995152	0.994659
top 16	0.999575	0.999314	0.999809	0.999422	0.995712	0.995249
top 17	0.999575	0.999328	0.999814	0.999675	0.995715	0.995379
top 18	0.999574	0.999319	0.999799	0.999427	0.995826	0.994986
top 19	0.999575	0.999315	0.999778	0.999386	0.994743	0.994461
top 20	0.999861	0.999423	0.999746	0.999299	0.993901	0.993674
top 21	0.999803	0.999404	0.999796	0.999645	0.994941	0.994920

Table S14. The Results of State-of-the-art and MiRAGE Methods on the B-Dataset.

Model	AUROC	AUPR	Accuracy	Precision	Recall	F1-score
DRHGCN	0.9092	0.9106	0.8268	0.8678	0.7711	0.8166
HINGRL	0.8845	0.8774	0.8035	0.8006	0.8084	0.8045
DRWBNCF	0.9004	0.9018	0.5991	0.9810	0.2021	0.3352
DDAGDL	0.8421	0.8315	0.7646	0.7616	0.7703	0.7659
AMDGT	0.9337	0.9309	0.8629	0.8614	0.8650	0.8632
MiRAGE	**0.9996**	**0.9996**	**0.9918**	**0.9917**	**0.9918**	**0.9917**

Table S15. The Results of State-of-the-art and MiRAGE Methods on the C-Dataset.

Model	AUROC	AUPR	Accuracy	Precision	Recall	F1-score
DRHGCN	0.9324	0.9427	0.8652	0.9192	0.8008	0.8559
HINGRL	0.9372	0.9457	0.8698	0.8851	0.8500	0.8672
DRWBNCF	0.9234	0.9419	0.8663	0.8984	0.8370	0.8612
DDAGDL	0.8693	0.8935	0.8168	0.7874	0.7721	0.7797
AMDGT	0.9681	0.9698	0.9062	0.8903	**0.9265**	0.9081
MiRAGE	**0.9733**	**0.9672**	**0.9209**	**0.9210**	0.9209	**0.9214**
